# Household financial burden of phenylketonuria and its impact on treatment in China: a cross-sectional study

**DOI:** 10.1007/s10545-016-9995-0

**Published:** 2016-11-10

**Authors:** Lin Wang, Hui Zou, Fang Ye, Kundi Wang, Xiaowen Li, Zhihua Chen, Jie Chen, Bingjuan Han, Weimin Yu, Chun He, Ming Shen

**Affiliations:** 1 Department of Preventive Health Care, China-Japan Friendship Hospital, Beijing, China; 2 Newborn Screening Center, Jinan Maternity and Child Care Hospital, Jinan, Shandong Province China; 3 Department of Pediatrics, China-Japan Friendship Hospital, No. 2, Yinghua East Street, Chaoyang District, Beijing, 100029 China; 4 Department of Nutrition, China-Japan Friendship Hospital, Beijing, China; 5 Clinical Research Institute, China-Japan Friendship Hospital, Beijing, China

## Abstract

**Background:**

Phenylketonuria (PKU) is a rare inborn disease, which, untreated, leading to severe neurobehavioral dysfunction. Considering its complexity, the management of PKU may bring a formidable economic burden to parents and caregivers. It is still unknown what the out-of-pocket expenses are for a patient with PKU in China. This paper explores the household financial burden of classical PKU and its impact on Chinese families in a quantitative manner for the first time.

**Methods:**

A non-interventional and observational study was conducted at the China-Japan Friendship Hospital, one of the national centers for inherited metabolic disorders in China. The medical and non-medical household financial burdens were consolidated into a questionnaire to evaluate the out-of-pocket costs (OOPCs) of PKU treatment and follow-up.

**Findings:**

The total OOPCs were USD$3766.1 (0y), USD$3795.2 (1–2 ys), USD$4657.7 (3–4 ys), USD$5979.9 (5–8 ys), and USD$5588.7 (9 ys and older) for PKU patients of different age groups. The median economic burden of classical PKU was 75.0 % of total annual family income (range 1.0–779.1 %), and 94.4 % of the families exceeding the threshold considered as catastrophic expenditure. There was a negative correlation between the financial burden and the proportion of time when Phe concentrations were in the desired target range (120–250 μmol/L) in 0–4-ys group (*r* = -0.474, *p* = 0.026).

**Conclusions:**

The management of PKU is associated with a severe financial burden on patients’ families, which may lead to insufficient treatment or variation of blood Phe concentration. The current reimbursement policies are as yet inadequate. A national reimbursement system targeting treatment practices for PKU patients and other rare diseases across China is imperative.

**Electronic supplementary material:**

The online version of this article (doi:10.1007/s10545-016-9995-0) contains supplementary material, which is available to authorized users.

## Background

Newborn screening program was introduced to China in 1981, when a pilot plan was developed to demonstrate the feasibility of its implementation (Blau et al 2010; Guthrie and Susi 1963). Since the introduction of the Law of Maternal and Infant Health Care in 1995, the program had been rapidly developed across the country (Chen et al 1983; Shi et al 2012). By the end of 2009, 179 centers in 30 provinces had conducted newborn screening for phenylketonuria (PKU, OMIM 261600) and congenital hypothyroidism (CH) (Gu et al 2008; Padilla and Therrell 2007; Cao et al 2009; Wang et al 2010). A series of national and local administrative measures were implemented in 2009 to diminish the geographic differences in the coverage of newborn screening (The technical specification of newborn screening; Mei et al 2013a, b). With the expansion of its coverage, the number of neonates screened increased from 5 million in 2006 to more than 10 million in 2013 (Cao et al 2009; Wang et al 2010; Ye et al 2013). According to the report of “The technical specification of newborn screening”, the proportion of screening was 87.98 %(total number of newborn was 16,714,051, the total number of screened was 14,705,320) in 2013.

As one of the rare diseases, PKU is an inborn error in amino acid metabolism which leads to profound neurobehavioral dysfunction. Early diagnosis and prompt intervention enables most patients to avoid the progression of mental disability. Restrictive diet remains the mainstay of therapy although new emerging options allow certain dietary freedom. However, the complex management of PKU may present a significant economic burden for parents or caregivers (Eijgelshoven et al 2013; Guest et al 2013). This burden includes direct costs which are usually associated with low protein foods, supplements, medications, laboratory monitoring, and indirect costs which mean caregivers’ lost of productivity. Costs and reimbursements of dietary therapy vary widely in different countries (Guest et al 2013). In most developed countries such as the United States, the European Union, and Japan, health insurance programs have included the expenditure on newborn screening. National Health Services or Medicaid also pay for families that are in need of treatment (http://www.nichd.nih.gov; American Academy of Pediatrics Committee on Nutrition 1994; Belanger-Quintana et al 2012; Kitagawa 2012; European society for phenylketonuria and allied disorders treated as phenylketonuria; Mei et al 2013a, b). Therefore, a vast majority of families do not have to pay for the treatment of PKU except minor out-of-pocket costs (OOPCs) incurred during the treatment.

In China, the annual costs for PKU treatment range between 15,000 and 25,000 RMB ¥ (2400–4000 USD$) approximately (http://www.gdnsn.com). However, no national reimbursement systems or health insurance programs have been set up to specifically cover the expenditures (Wang et al 2010). Only a few local governments or newborn screening centers in Beijing, Shanghai, Shandong, and Guangdong are able to support the treatment of children under 6–18 years of age by providing part of phenylalanine (Phe)-free formulas or cash reimbursement (The technical specification of newborn screening; Mei et al 2013a, b; http://www.gdnsn.com). Other expenditures such as low-protein foods, supplements, medications, laboratory monitoring, transportation, and healthcare visits have to be covered by the patients themselves. Therefore, the major expenses are actually incurred by patients’ OOPCs. Due to the high cost of tetrahydropterin (BH_4_), almost none of the BH_4_-responsive patients can afford it.

Although data on the healthcare burden of PKU in developed countries are available, studies on personal economic burden, as well as the strategies used especially by poor families to pay for treatment-related expenses, and the impact on families with PKU patients, are inadequate in China. Therefore, we have conducted this study to identify the OOPCs of PKU, and its economic burden on households in China. Because of the minor impact and therefore lower costs of mild PKU on patients and family, this study focused on the classical PKU, which is also the most common manifestation.

## Methods

### Study design and participants

A non-interventional, observational study was conducted between July 2014 and June 2015 at the China-Japan Friendship Hospital, one of the national centers for inherited metabolic disease in China. Families afflicted with classical PKU were enrolled during their regular outpatient examination based on the following inclusion criteria:

1. Diagnosis of classical PKU (untreated Phe level >1200 μmol/L) (Blau et al 2010; Lindner 2006; Scriver et al 1995);
2. Uninterrupted dietary treatment in the past year;
3. Families from 20 provinces and municipalities with prevalence of PKU higher than 1:20,000 (“The technical specification of newborn screening”, Supplementary Table 1).

A total of 170 patients with classical PKU attended our hospital between July 2014 and June 2015, of these 165 were enrolled in this study. After excluding 38 cases for different reasons listed in Supplementary Fig. 1, 127 cases are finally included in the results.

The study protocol and data collection instruments were approved by the ethical review boards of China-Japan Friendship Hospital. Signed informed consent was obtained prior to any interview.

### Data collection

The patient’s caregivers were invited for a face-to-face interview immediately after routine examination. Each interview was conducted by members of the research team who had received standardized training. We used a systematic literature based quantitative questionnaire to collect all the economic cost from the patients and their families. The findings of the literature review were confirmed through interviews with three opinion leaders in the field of PKU, who detailed the various costs associated with the management of PKU in China. This ensured that all potential OOPCs possibly associated with living with PKU were inclusive in the study questionnaires. Items included costs of living with PKU, such as medications for primary and differential diagnosis, treatment costs in different age groups using domestic or imported Phe-free formulas, low-protein foods, supplements and medications, costs for regular laboratory monitoring, special training and healthcare visits (approximately every 1–2 months. Patients who lived very far from Beijing sent dry spot paper to our laboratory every month). Data were categorized into subgroups according to age, medical and non-medical costs, with or without governmental support. This enabled assessment of the varying burden of PKU in different patients’ characteristics.

### Statistical analysis

The database was developed by Epidata 3.1 software and the statistical analyses were conducted with SPSS Version 15.0. The demographic characteristics of patients and their parents were collected and analyzed in contrast with other characteristics. Categorical variables were described as the percentage of the total sample size. Continuous variables were described by median (interquartile ranges) and mean ± SD (standard deviation). Differences between subgroups were compared for statistical difference using Kruskal-Wallis analysis for variables following skewed distribution. Sensitivity analyses comparing the key indicator before and after removing outliers higher than ±3 SDs of mean were performed. Spearman analysis was conducted to examine the correlation between skew-distributed variables and potential influencing factors. *P* < 0.05 was considered statistically significant. The average exchange rate in December 2014 (USD$1 = ¥6.2) was used to convert the money from Chinese RMB ¥ to USD$.

## Results

### Study samples

A total of 165 classical PKU patients and their families were recruited, 127(77.0 %) of which accepted the interview and completed the questionnaire. The age, gender, and recent plasma phenylalanine levels were not significantly different between families irrespective of interview acceptance. The patients and their families originated from 20 provinces and municipalities in China as outlined above.

All 127 patients were treated with a protein-restricted diet, including Phe-free formulas and low-protein rice or flour, and followed by regular monitoring of plasma phenylalanine.

The parent-participants were the main caregivers although two patients were orphans and their parent-related information was unavailable. Detailed clinical demographics are listed in Table 1.

**Table 1:** Clinical demographics of classical PKU

Demographic characteristics	Classical PKU (*n* = 127)
Patients	
Median age in months of patients (range in months) ^a^	53.0 (1.0–270.0)
% of female patients	47.2
Median age in months of PKU diagnosis (range in months) ^a^	1.0 (0.0–152.0)
% of diagnosis by newborn screening	65.9
Median of latest plasma Phe concentration (range in mg/dl)	5.30 (0.22–26.95)
% of siblings affected by PKU	22.2
Parents	
Median age of fathers (range in years) ^a^	30.5 (27.0–35.0)
Median age of mothers (range in years) ^a^	29.0 (25.0–33.0)
% of fathers educated more than 9 ys ^a^	48.0
% of mothers educated more than 9 ys ^a^	48.8
% of nuclear families^b^	44.1
% of rural families	77.2
Median of annual income per capita (USD$) (Interquartile range)^c^	1613 (806–2688)
Percentage of patients that was designed to benefit from reimbursement policies	75.6
Percentage of patients that had actually obtained the reimbursements	29.9

^a^ *n* = 125 with two missing values because the patients were orphans. One of the orphans was raised by an orphanage, the other one was adopted by another family

^b^nulear family: the couple with children, but no other family members

^c^USD$ 1 = RMB¥ 6.2 in December 2014

### OOPCs for afflicted families and influencing factors

Table 2 lists the direct costs related to PKU, including medical and non-medical costs. The mean total direct cost was USD$ 5833.6 (median USD$ 4798.4), with Phe-free formulas as the main component (58.3 %).

**Table 2:** Total costs in patients of classical PKU (USD$)^a^

Direct costs	Mean ± SD	% in total direct costs	Median (IQR)
Medical costs	1612.4 ± 6383.2	27.9	508.1 (411.3–701.6)
Medical examination^b^	694.1 ± 1129.0	11.9	469.4 (382.3–604.8)
Rehabilitation treatment	918.2 ± 5621.9	16.0	0.0 (0.0–0.0)
Non-medical costs^c^	4221.2 ± 2135.5	72.1	4225.8 (2618.1–5838.7)
Phe-free formulas	3421.7 ± 1902.0	58.3	3096.8 (1935.5–4838.7)
Domestic fomulas	3169.5 ± 1641.2	–	2817.3 (1935.5–3819.3)
Imported fomulas	4427.0 ± 2346.5	–	4206.4 (3831.6–4838.7)
Low-protein rice and flour, etc·	572.5 ± 418.2	9.9	580.6 (290.3–774.2)
Extra costs (accommodation, transportation for medical treatment)	227.0 ± 412.6	3.9	48.4 (0.0–251.6)
Total direct costs	5833.6 ± 6926.2	100.0	4798.4 (3551.9–6508.1)

^a^USD$ 1 = RMB¥ 6.2 in December 2014

^b^Including genetic tests, routine blood examination, serum phenylalanine, blood test for liver and renal function, serum lipids, and IQ screening

^c^Including Phe-free formulas, low-protein rice and flour, etc., and extra costs (accommodation, transportation for medical treatment)

After subtracting expenses that were reimbursed by the local governments or newborn screening centers, we found that the remaining total OOPCs were USD$3766.1 (0 y), USD$3795.2 (1–2 ys), USD$4657.7 (3–4 ys), USD$5979.9 (5–8 ys), and USD$5588.7 (9 ys and older). Results from Spearman correlation analysis indicated a positive correlation between the total direct costs and patient’s age with statistical significance (*r* = 0.366, *p* < 0.001).

### Financial burden and catastrophic expenditure

The World Bank has characterized economic burden as a ratio of direct cost to the total annual income of the household, and the burden becomes catastrophic if the ratio exceeds 10.0 % (Prescott 1999; Ranson 2002). Our data showed that the median economic burden of classical PKU was 75.0 % (range 1.0–779.1 %) and 119 of 126 (94.4 %) families suffered from catastrophic expenditure. The distribution of the ratios were as follows: 0.0–0.099 (5.6 % of households), 0.1–0.399 (11.9 % of households), 0.4–0.599 (21.4 % of households), 0.6–0.799 (16.7 % of households), 0.8–0.999 (11.1 % of households), 1.0–1.999 (15.1 % of households), 2.0–2.999 (5.6 % of households), 3.0–4.999 (8.7 % of households), higher than 5.0 (4.0 % of households).

Furthermore, Spearman correlation analysis showed that a lower individual family income was correlated with significantly higher economic burden (*r* = -0.269, *p* = 0.009).

### Financial burden and blood phenylalanine control

When we evaluated correlations between the financial burden and Phe concentrations, it became obvious that mean blood Phe for the entire history did not correlate with the economic status and burden irrespective of age groups. None of the standard deviations (SDs) for Phe concentrations correlated significantly with burdens of any age groups. Then the proportion of time when Phe concentrations were in the target range were investigated and found to correlate significantly and negatively with the financial burden in the 0–4-ys group (*r* = -0.474, *p* = 0.026), but not in the ≥5-ys group. With respect to higher thresholds for Phe concentrations, the correlations of financial burden and the proportion of time when Phe concentrations were >250 or 400 μmol/L were not significantly correlated (shown in Table 3).

**Table 3:** Correlation between household financial burden and percentage of blood Phe concentration

Age groups of patients		Financial burden in patients between 0 and 4ys		Financial burden in patients ≥5ys	
		r	p	r	p
Mean phe concentration		0.036	0.915	0.349	0.143
SD of phe concentration		0.427	0.190	−0.081	0.743
Proportion of time when Phe concentrations	in 120–250 μmol/L	−0.474	0.026	−0.190^a^	0.436
	>250 μmol/L	0.394	0.069		
	>400 μmol/L	0.349	0.293	0.190	0.436

^a^The desired target range of Phe concentrations were within 120–250 μmol in group of <4-ys old, <400 μmol in >5-ys groups

### Sensitivity analysis

The total direct costs were one of the key indicators in this study. We compared the costs with and without 3 outliers higher than ± 2 SDs of mean. The total direct cost was USD$ 5833.6 ± 6926.2 vs. USD$ 4919.1 ± 2381.0 (median USD$ 4798.4 vs. USD$ 4751.6). And the mean economic burden was 132.5 % vs. 119.1 % (median 75.0 % vs. 73.0 %).

### Reimbursement policies across the country

**Fig. 1:**
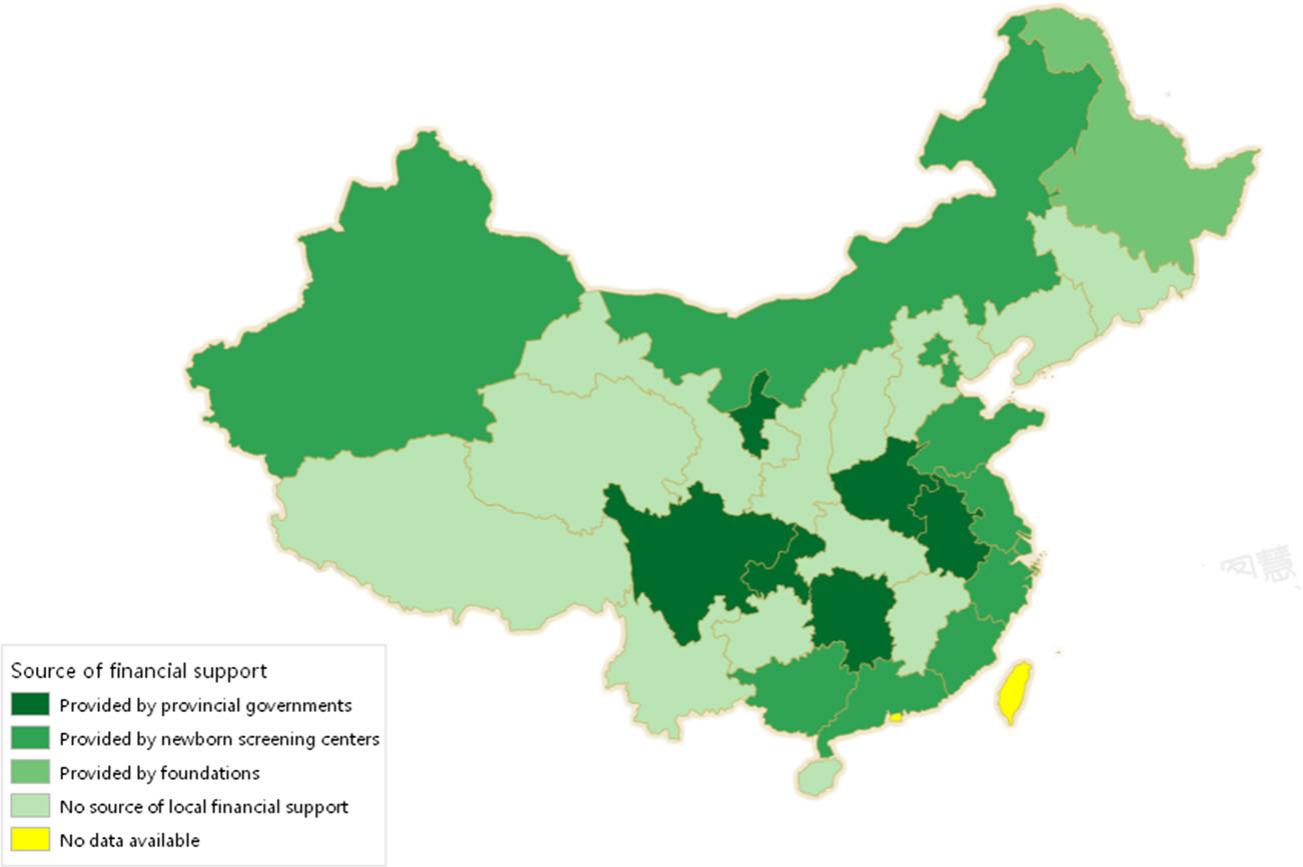
Source of financial support in different provinces in China

Besides data from the 20 provinces and municipalities, we also obtained data of ten more provinces by interviewing the staff on telephone who were in charge of the refund policies. All the data across the country are shown in Fig. 1. This map identifies three different sources and levels of support. The first and also the best support was provided by the provincial government up to 18 years of age in Henan, Ningxia, Anhui, Hunan, Chongqing, and Sichuan provinces. The patients could obtain free domestic formulas from the government every 3 months to 1 year which could cover two thirds of their annual expenditures. This aid was steady and enabled the affected families to meet most of their expenses (NHFPC. The technical specification of newborn screening; http://www.gdnsn.com; http://www.ahhfld.gov.cn; Yang et al 2011). The second best local support was provided by the provincial capital’s newborn screening centers from Beijing, Tianjin, Shandong, Inner Mongolia, Xinjiang, Zhejiang, Shanghai, Fujian, Jiangsu, Guangxi and Guangdong, where patients could receive money refund or free domestic formulas. The money is about USD$1290 to 2420 per year which account to half to two thirds of their annual expenditures. The third source were foundations, such as the Red Cross Foundation of Heilongjiang province, offering financial aid to patients up to 6 years of age. Almost no specialized provincial financial supports were available in the rest of the provinces (Qinghai, Gansu, Tibet, Shanxi, Shaanxi, Hebei, Jilin, Liaoning, Hubei, Guizhou, Yunnan, Jiangxi, and Hainan).

## Discussion

To our knowledge, this is the first study to investigate the overall individual medical and non-medical financial burden of families with PKU in China. With the increasing coverage of newborn screening, the biggest challenge has now become to ensure the quality of treatment, and minimize its impact on the quality of life of patients and their families. A total of 127 patients and their parents/caregivers from 20 provinces and municipalities were interviewed to investigate the actual expenses for each family. The findings indicated that the total expenditure for treatment was significantly higher than in previous reports in China (Lindner 2006; Scriver et al 1995). Excluding expenses that were reimbursed by the government, the median OOPCs were USD$4798.4 (3551.9–6508.1), much higher than that in western countries. In the Netherlands, the OOPC was reported only €604 (€28–1206) (USD$794, USD$37-1587, USD$1 = €0.76 in 2013) annually including medical and non-medical costs (Eijgelshoven et al 2013). As previous reported, all costs on protein substitutes were either fully reimbursed or covered by government and state monetary benefits while the mean annual cost was still high ranging from €4273 to €21,590 (USD$5583 to USD$28408, USD$1 = €0.76 in 2013) per patient in ten European countries (Belanger-Quintana et al 2012; Guest et al 2013) .

According to the World Bank (Prescott 1999; Ranson 2002), economic burden exceeding 10 % results in adverse effects on daily life. The high proportion (94.4 %) of families who had catastrophic health expenditures as a result of trying to care for a PKU patient derived financial support from grandparents, loans, personal savings or sale of assets—thereby driving poor households even deeper into poverty. The overall magnitude of the economic shock requires serious attention by local and provincial authorities. Households with catastrophic economic burden coupled with fewer assets, reduced savings, and increased debt are likely to suffer consequences that affect all family members. The consequences include reduced expenditures for food and education, as well as the choice of treatment.

Results showed that households with higher economic burden sustained poorer control of blood phenylalanine concentration in desired target range of 120–250 μmol/L in 0–4-ys group, which demonstrated that severe financial burden may lead to insufficient treatment especially in the early years of treatment. Although other factors influencing Phe concentrations cannot be excluded, the present study highlights the importance of relieving economic burden for better management of PKU in China, especially in the first 4 years. In addition, a small number of patients were even left untreated, stopped treatment very early or abandoned because of the sudden economic shock on families of low socioeconomic level (The technical specification of newborn screening; Yang et al 2011; http://www.moh.gov.cn). Legislation for mandatory care of patients via increased financial aid in China is imperative (Gao et al 2013). As clearly stated, there is “no logic of mandating screening without also providing for treatment” (Buist and Huntington 2007).

The local governmental support with dietary formulas and cash compensation to the afflicted families partially alleviates the economic shock of high treatment costs. Nearly a third (29.9 %) of the afflicted families reported reimbursement after they provided evidence of treatment-related expenses. However, the financial relief was associated with several challenges, the most important of which is related to formula support limited to domestic products with less treatment compliance because of unfavorable flavor or taste. Most imported formulas, other foods and supplements had to be exclusively paid by patients and families themselves. In some provinces, support failed to adapt with aging, resulting in inadequate supply of formula or cash reimbursements for growing patients. Moreover, the reimbursement was often delayed by half up to 1 year and failed to meet the immediate needs of patients, thereby increasing the OOPCs of parents.

The absence of a national government reimbursement system and mandatory regulations for treatment in China precludes long-term treatment or follow-up of PKU. The Chinese government, therefore, started a pilot program known as the New Rural Co-operative Medical System (NRCMS) to help rural kids under 10 years of age by reimbursing their medical expenses in January 2013 (The technical specification of newborn screening; http://www.moh.gov.cn). However, the program did not materialize with the PKU treatment as the special formula in China are not listed in the drug formulary. In addition, a significant number of patients who had migrated to large cities to earn their living with their parents were disqualified for reimbursement in their hometown as well as in large cities. As a result, despite the large coverage indicated by the results in Table 1, only a small number of patients could benefit from the NRCMS (75.6 % v.s. 29.9 %). This fact has highlighted the gap between the great intention of policy-makers and disappointing results actually achieved.

We hope that our study will encourage other researchers to advocate effectively for increased financial support for poor families in China, afflicted with PKU. Furthermore, PKU is only one prominent example for the challenges that families with children suffering from orphan diseases have to face. Even fewer families can afford the treatment for BH_4_-responsive patients due to the absence of domestic drugs and financial support.

According to Dr. Gu and colleagues the prevalence of detectable rare disorders in China was one in 5800. As a country with a huge population of 1.3 billion, approximately 200,000 newborns with rare diseases are born in China each year (Gu et al 2008; Wang et al 2010). Most of them face the difficulty in dealing with costs of diagnostics and specialized treatments such as expensive dietary treatment or alternative drugs from abroad, e.g., for patients with methylmalonic academia, glutaric academiaI or lysosomal storage diseases, etc. Several non-governmental organizations (NGOs) specialized in rare diseases were founded to support the patients in health care, daily life, and advocacy, such as the China PKU Alliance, China-Dolls Care and Support Association, etc., however, these NGOs cannot be the main power in the financial assistance (Wang et al 2010). The national and local governments should re-examine which effective policies or means of support are necessary to families who have experienced catastrophic health expenditures. Financial assistance by national health services or foundations, new types of insurance, special public subsidies for PKU and other orphan diseases are needed including prompt funding and emergency financial relief. The treatment of PKU is far from optimal in wider parts of the world, including China. There is a clear need for greater uniformity in the national reimbursement system targeting patients with PKU across the whole country. Easy access, greater aid, and regionally qualified dieticians and nutritionists, are urgently needed to improve patients’ conditions, outcome, and quality of life. Studies with long-term follow-up of therapeutic outcomes and efficacy results, focusing on life quality of affected families, especially the parents/caregivers, are needed to fully understand and tackle the economic impact of PKU on families.

### Limitations

This paper presents exploratory analyses of actual costs and impact on the family life of PKU patients in China. Due to the exploratory nature of the study, the findings should be interpreted with caution.

Due to the relatively small sample size in some provinces, the data of these provinces may be less representative. To improve it, we interviewed the staff in charge of PKU reimbursement policies in these provinces by telephone. The information they provided was in accord with the data we collected, which proved that the data were correct and unbiased. Nevertheless, a more extensive multi-center study is needed to investigate the economic burden in China more comprehensively.

Notably, the most affected families in this study incurred expenses traveling to Beijing for medical intervention in a national inherited metabolic disorders center, which might have introduced a bias compared with other families treated at local hospitals. However, travel expenses were only 3.9 % of the total direct costs, which minimally affected the final results.

## Conclusions

The management of PKU is associated with a severe financial burden on patients’ families. The current reimbursement policies is inadequate. A national reimbursement system targeting treatment practices for patients with PKU as well as other rare diseases across China is imperative.

Abbreviations *PKU* Phenylketonuria, *OOPCs* Out-of-pocket costs, *CH* Congenital hypothyroidism, *BH4* Tetrahydropterin, *SD* Standard deviation, *NRCMS* New Rural Co-operative Medical System, *NGOs* Non-governmental organizations

## Electronic supplementary material

Below is the link to the electronic supplementary material.

ESM 1 (GIF 30 kb)

High Resolution Image (TIF 252 kb)

ESM 2 (GIF 9 kb)

High Resolution Image (TIF 94 kb)

ESM 3 (DOCX 15 kb)
