# Supplementary figures and images for: Household financial burden of phenylketonuria and its impact on treatment in China: a cross-sectional study
Source: J Inherit Metab Dis. 2016 Nov 10;40(3):369–76. doi: 10.1007/s10545-016-9995-0 (PMC5393103; doi:10.1007/s10545-016-9995-0)

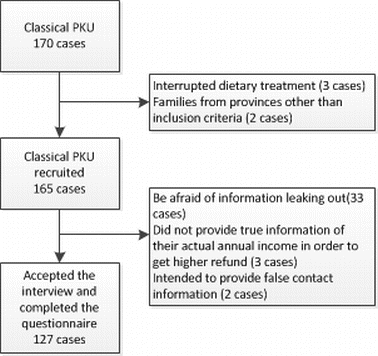

Supplement: Supplementary file 1 — (GIF 30 kb) [file 10545_2016_9995_Fig2_ESM.gif]

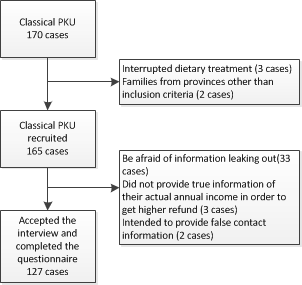

Supplement: Supplementary file 2 — High Resolution Image (TIF 252 kb) [file 10545_2016_9995_MOESM1_ESM.tif]

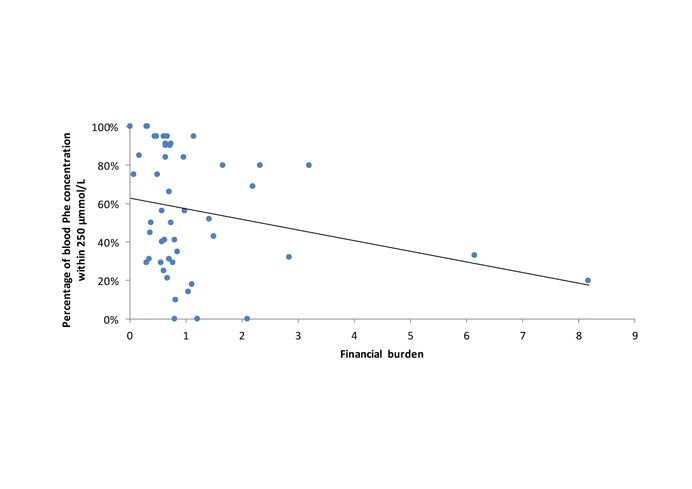

Supplement: Supplementary file 3 — (GIF 9 kb) [file 10545_2016_9995_Fig3_ESM.gif]

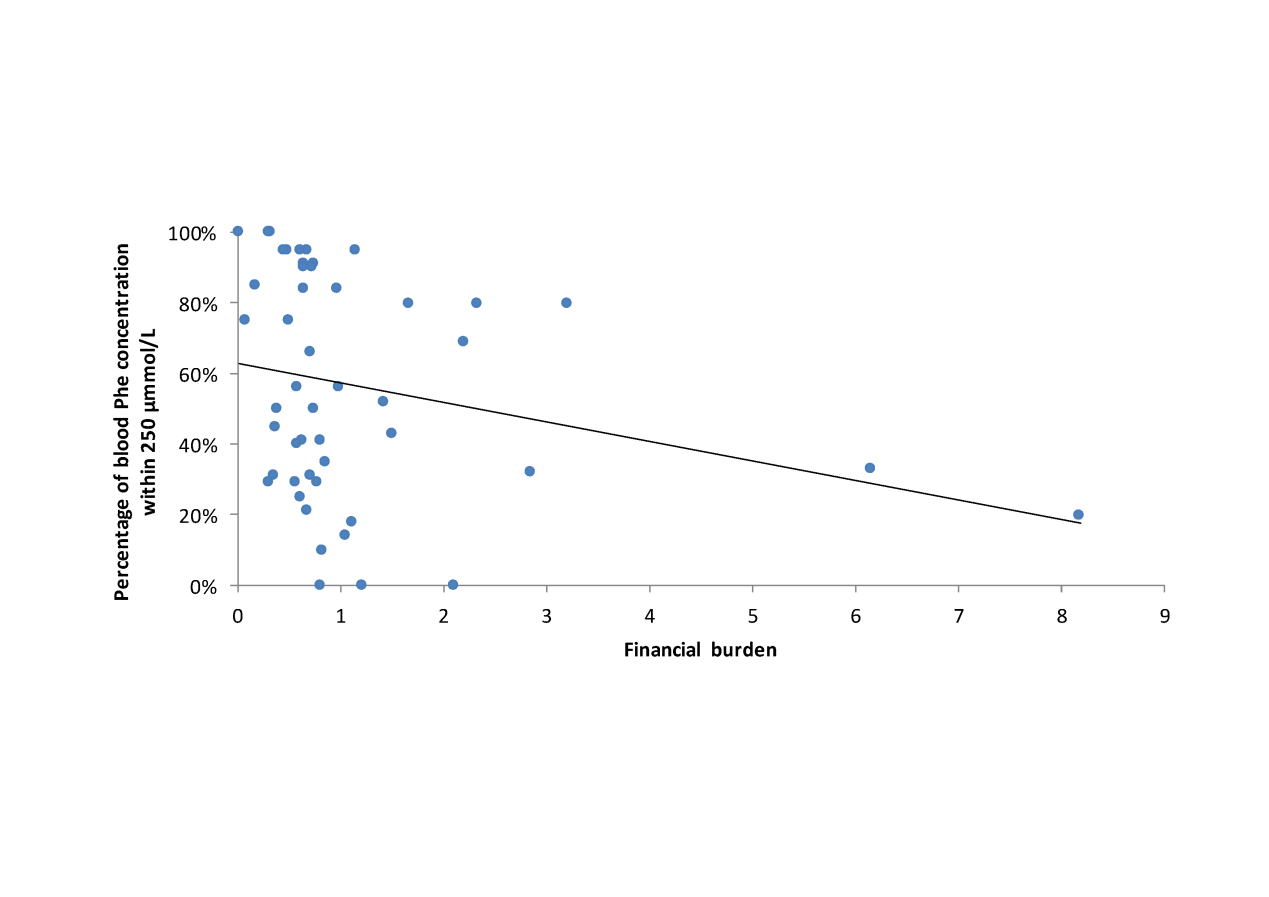

Supplement: Supplementary file 4 — High Resolution Image (TIF 94 kb) [file 10545_2016_9995_MOESM2_ESM.tif]
