# Supplementary material for: Household financial burden of phenylketonuria and its impact on treatment in China: a cross-sectional study
Source: J Inherit Metab Dis. 2016 Nov 10;40(3):369–76. doi: 10.1007/s10545-016-9995-0 (PMC5393103; doi:10.1007/s10545-016-9995-0)
Supplement: Supplementary file 5 — (DOCX 15 kb) [file 10545_2016_9995_MOESM3_ESM.docx]

Supplementary Table 1. Provinces and municipalities with prevalence of PKU higher than 1:20,000

| Province / Municipality | Incidence |
| --- | --- |
| Beijing | 1/10287 |
| Tianjin | 1/6744 |
| Liaoning | 1/8726 |
| Shanghai | 1/13871 |
| Jiangsu | 1/12899 |
| Shandong | 1/9920 |
| Hebei | 1/9271 |
| Shanxi | 1/5752 |
| Jilin | 1/7942 |
| Heilongjiang | 1/9012 |
| Anhui | 1/13156 |
| Henan | 1/11381 |
| Hubei | 1/19585 |
| Inner Mongolia | 1/12396 |
| Yunnan | 1/19234 |
| Shaanxi | 1/10097 |
| Gansu | 1/5900 |
| Qinghai | 1/5108 |
| Ningxia | 1/4893 |
| Xinjiang | 1/7001 |
